# Ultrasound-Guided Injections of HYADD4 for Knee Osteoarthritis Improves Pain and Functional Outcomes at 3, 6, and 12 Months without Changes in Measured Synovial Fluid, Serum Collagen Biomarkers, or Most Synovial Fluid Biomarker Proteins at 3 Months

**DOI:** 10.3390/jcm12175541

**Published:** 2023-08-25

**Authors:** Richard T. Meehan, Mary T. Gill, Eric D. Hoffman, Claire M. Coeshott, Manuel D. Galvan, Molly L. Wolf, Isabelle A. Amigues, Liudmila M. Kastsianok, Elizabeth A. Regan, James L. Crooks, Gregory J. Czuczman, Vijaya Knight

**Affiliations:** 1 Departments of Medicine, Clinical Labs, Radiology and Divisions of Rheumatology, Immunology/Complement Labs, and Biostatistics and Bioinformatics, National Jewish Health, Denver, CO 80206, USA; gillm@njhealth.org (M.T.G.); ehoffman@arthroventions.org (E.D.H.); coeshottc@njhealth.org (C.M.C.); galvanm@njhealth.org (M.D.G.); molly.wolf@vailhealth.org (M.L.W.); dramigues@unabridgedmd.com (I.A.A.); regane@njhealth.org (E.A.R.); crooksj@njhealth.org (J.L.C.); gregory.czuzcman@riao.com (G.J.C.); 2 Department of Epidemiology, Colorado School of Public Health, CU Anschutz School of Medicine, University of Colorado, Aurora, CO 80045, USA; 3 Radiology Imaging Associates, Englewood, CO 80112, USA; 4 Department of Pediatrics, Section of Allergy and Immunology, CU Anschutz School of Medicine, University of Colorado, Aurora, CO 80045, USA; vijaya.knight@childrenscolorado.org

**Keywords:** osteoarthritis, ultrasound, synovial fluid, biomarkers, cytokines

## Abstract

Background: Prior studies have demonstrated improved efficacy when intra-articular (IA) therapeutics are injected using ultrasound (US) guidance. The aim of this study was to determine if clinical improvement in pain and function after IA hyaluronic acid injections using US is associated with changes in SF volumes and biomarker proteins at 3 months. Methods: 49 subjects with symptomatic knee OA, BMI < 40, and KL radiographic grade II or III participated. Subjects with adequate aspirated synovial fluid (SF) volumes received two US-guided IA-HA injections of HYADD4 (24 mg/3 mL) 7 days apart. Clinical evaluations at 3, 6, and 12 months included WOMAC, VAS, PCS scores, 6 MWD, and US-measured SF depth. SF and blood were collected at 3 months and analyzed for four serum OA biomarkers and fifteen SF proteins. Results: Statistical differences were observed at 3, 6, and 12 months compared to baseline values, with improvements at 12 months for WOMAC scores (50%), VAS (54%), and PCS scores (24%). MMP10 levels were lower at 3 months without changes in SF volumes, serum levels of C2C, COMP, HA, CPII, or SF levels of IL-1 ra, IL-4, 6, 7, 8, 15, 18, ILGFBP-1, 3, and MMP 1, 2, 3, 8, 9. Baseline clinical features or SF biomarker protein levels did not predict responsiveness at 3 months. Conclusions: Clinical improvements were observed at 12 months using US needle guidance for IA HA, whereas only one SF protein biomarker protein was different at 3 months. Larger studies are needed to identify which SF biomarkers will predict which individual OA patients will receive the greatest benefit from IA therapeutics.

## 1. Introduction

Knee osteoarthritis (OA) is an increasingly common leading cause of disability and is costly to manage, especially if patients progress and require surgical intervention [1,2]. Unfortunately, there are no effective FDA-approved disease-modifying therapeutic agents that can halt or reverse cartilage loss in knee OA. However, two recently published large cardiovascular treatment trials demonstrated that inhibition of IL-1 beta by canakinumab and low-dose colchicine reduced the incidence of hip and knee arthroplasty compared to the placebo groups [3,4]. Therefore, intra-articular (IA) hyaluronic acid (HA) injections are common treatment options for knee OA patients to provide symptomatic pain relief or for those who are not surgical candidates or choose to defer total joint arthroplasty [5].

Recent studies emphasize the knee as an organ containing important supporting structures, including the subchondral bone, ligaments, joint capsule, meniscus, synovium, and the surrounding musculature, in addition to cartilage [6]. There is also growing evidence that knee OA is associated with chronic inflammation and phenotypes rather than a non-inflammatory degenerative joint disease process. Synovial biopsies revealed synovitis in 50% of patients with early OA, and synovial fluid (SF) analysis revealed cytokines and other inflammatory mediators that distinguish early from advanced knee OA [7,8]. The presence of substantial synovial effusion and synovitis on MRI also correlates with subsequent loss of knee cartilage among OA patients [9]. It has also been reported that OA SF contains a pro-inflammatory cytokine profile [10,11,12,13,14]. We also observed that many OA patients with sufficiently severe knee pain requesting an IA glucocorticoid or HA injection have a pro-inflammatory SF cytokine profile similar to that of many rheumatoid arthritis (RA) patients [15]. SF is also a rich source of potentially valuable biomarkers that may be used to classify different OA endotypes and, hopefully, in the future, may help guide therapy by predicting drug responsiveness for individual patients [16].

There are multiple proposed mechanisms whereby IA HA injections might provide clinical benefit in OA [17,18,19]. These include anti-inflammatory properties based upon in vitro studies, improved SF viscoelastic, rheologic, and frictional properties of HA, and possible chondroprotection since HA also interacts directly with articular cartilage [20].

The viscosupplement used in this study, HYADD4 (Hymovis^®^ Fidia, Abano Terme, Padova, Italy), is a modified derivative of HA with a molecular weight of 500–730 kDa obtained by a controlled chemical–physical synthesis process with 2% of the carboxyl radicals on the glucuronic acid present in the polysaccharide chain conjugated with an aliphatic amine (hexadexyclamine) [21]. The chemical modification of HA by the addition of hexadecylamine increases the rheological properties of HYADD4, conferring higher viscoelasticity in solution compared to other HA derivatives of the same molecular weight. To confirm delivery of this HA product into the synovial fluid compartment, we utilized ultrasound (US) guidance with direct needle visualization during all injections and aspirations. Other studies have confirmed that US delivery of glucocorticoids into the knee joint space is more effective and less painful than non-image-based injections [22,23].

We chose to use direct needle visualization with US guidance as well as an external pneumatic compression device to enhance the success of aspiration even in patients with very small SF effusions since no OA patients were excluded from this study based upon the size of knee effusions on US [24]. To our knowledge, this is the first study to determine if an IA HA injection alters SF volumes as a surrogate for intra-articular inflammation and if baseline clinical features or SF protein levels predict clinical responsiveness at 3 months when HA is delivered with US guidance.

## 2. Materials and Methods

### 2.1. Subjects

This single-center, open-label, prospective, investigator-initiated knee OA biomarker study (HS 3179, ClinicalTrials.gov accessed on 5 July 2023 NCT 04093232) was conducted with all subjects providing informed consent after Institutional Review Board approval. We recruited subjects from National Jewish Health (NJH) clinics, clinical trial notification web sites, and local radio advertisements. Exclusion criteria included age < 21 or >80 years, BMI > 40, pregnancy, knee surgery within one year, IA injectable therapeutics within 3 months, a history of systemic inflammatory or crystal arthritis, prior allergic reactions to chloroprep, lidocaine, or HA products, or any use of oral or systemic immunomodulatory therapeutics. Subjects were also required to ambulate for 6 min without the use of walking assistive devices, and the diagnosis of knee osteoarthritis was confirmed by a NJH study rheumatologist. Weight-bearing tibiofemoral joint radiographs were obtained within 1 year of their first study visit. These images were reviewed by a fellowship-trained musculoskeletal (MSK) radiologist for study inclusion based upon the presence of Kellgren-Lawrence (KL) grade II or III osteoarthritis [25]. Forty-nine eligible subjects had one knee aspirated between 2019 and 2021, and if an adequate SF volume of ≥0.5 mL was aspirated, they received the first of two intra-articular (IA) injections of an HA HYADD 4 (Hymovis^®^ 24 mg/3 mL, Fidia Farmaceutici S.p.A., Abano Terme, Padova, Italy) and provided simultaneous peripheral blood samples.

The study protocol included five visits over 12 months. Baseline visit 1 included clinical assessments, knee aspiration, and an IA HA injection if the SF aspirated volume was ≥0.5 mL. Visit 2 was scheduled 7 days later for a second US-guided IA HA injection and a peripheral blood draw. Three additional visits at 3, 6, and 12 months were for clinical assessments and US-measured SF depth. 

### 2.2. Aspiration and Injection Technique

**Figure 1:**
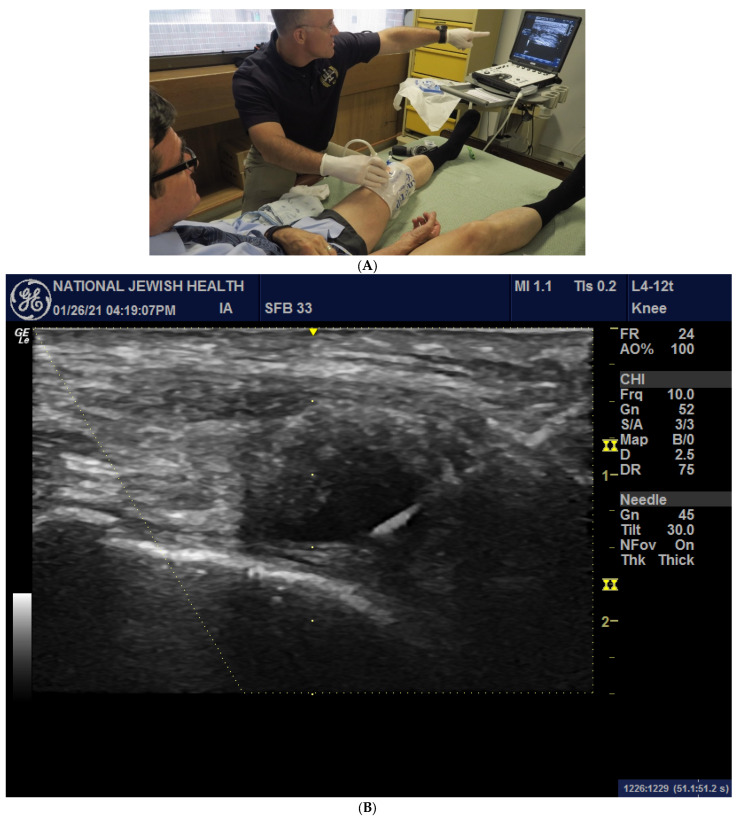
(**A**) An inflated pneumatic compression device with an image displayed on an ultrasound screen prior to successful knee aspiration of synovial fluid. Image courtesy of Dr. R. Meehan and Dr. R. Scheuring. (**B**) US image of a study subject during needle insertion, displaying a bright 20-gauge needle entering from the upper right-hand corner of the image with the tip placed within the intra-synovial space (dark anechoic region) during inflation and prior to injecting IA HA product.

An external pneumatic compression device (KneeTap^TM^ Arthroventions LLC, Denver, CO, USA) was inflated to 100 mmHg as previously described [24]. Ultrasound images were acquired using a GE LOGIQ e ultrasound (Fairfield, CT, USA) with a 12L-RS linear array probe, as displayed in Figure 1A,B.

A direct-in-line needle visualization technique was used for all procedures. The probe was covered with gel; a sterile sleeve (CIV-FlexTM Transducer Cover, CIVCO Kalona, IA, USA) and sterile gel were next applied. The injection site was cleansed with ChloroPrep One-Step (2% *w*/*v* chlorhexidine gluconate and 70% *v*/*v* isopropyl alcohol, Care Fusion, El Paso, TX, USA), and then a sterile drape was placed. All procedures were performed by an American College of Rheumatology (ACR) certified MSK US-trained rheumatologist who used sterile gloves, head coverings, and surgical face masks to reduce the risk of needle entry site contamination and per NJH COVID precaution guidelines. The superior-lateral site was selected most often with the knee in slight flexion during supine positioning. The needle entry site was selected based on the US location of the largest anechoic region in SF. The skin, joint capsule, and anechoic region were then infiltrated with 1–2 mLs of preservative-free 2% lidocaine HCL (40 mg/2 mL) without epinephrine (Hospira Inc., Lake Forest, IL, USA) using a 27-gauge needle with US visualization. Next, also with direct needle visualization, an 18-gauge needle on a syringe was advanced into the anesthetized region to avoid needle tip placement into the joint capsule, synovium, or plica during aspiration and injection. The steer needle image enhancement software program on the GE US instrument was utilized, which allowed visualization of the 27-gauge needle during local infiltration of lidocaine as well as the aspirating and injecting needle during product instillation. If an adequate amount of SF volume was obtained during visit 1, then with the 18-gauge needle remaining in place, HYADD4 (24 mg/3 mL) was injected under direct US visualization. For study visit 2, scheduled for 7 days later, a 20-gauge needle was placed on the IA HA syringe for direct US-visualized injection after local infiltration of lidocaine without an aspiration.

### 2.3. Sample Preparation and Analysis of SF and Serum Proteins

SF was aspirated into a 5 or 20 mL syringe (Medline Industries Inc., Mundelein, IL, USA) and rapidly transferred into 6 mL plastic tubes containing sodium heparin (BD, Becton Drive, Franklin Lakes, NJ, USA). SF white blood cells (WBCs) were counted on a Beckman Coulter ACT 2 diff hematology analyzer (Beckman Coulter, Loveland, CO, USA). Peripheral blood was then collected into 6 mL plastic vacutainer tubes (BD) without anticoagulant for serum samples. SF and peripheral blood samples were centrifuged at 2000 rpm for 10 min within 45 min of collection and then aliquoted into 200 µL or 50 µL vials for storage at −80 °C until analyzed. All SF analytes were measured by multiplex fluorescent bead (Luminex) immune assays using three separate R&D Systems Inc. kits (Minneapolis, MN, USA). The following analytes were quantitated in pg/mL: IL-1ra (Interleukin 1 receptor antagonist), IL-4 (Interleukin 4), IL-6 (Interleukin 6), IL-7 (Interleukin 7), IL-8 (Interleukin 8), IL-15 (Interleukin 15), IL-18 (Interleukin 18), IGFBP-1 (Insulin-Like Growth Factor Binding Protein 1), IGFBP-3 (Insulin-Like Growth Factor Binding Protein 3), and MMPs (Matrix Metalloproteinases) 1, 2, 3, 8, 9, and 10. The bead multiplex assay was performed as previously described [15]. Cytokine concentrations were calculated with reference to the standard curve for each analyte. 

Serum cartilage biomarkers were analyzed in the Duke University Molecular Physiology Institute laboratory (Durham, NC, USA), under the direction of Virginia Kraus, MD, PhD. These included Collagen Type II cleavage product (C2C), Hyaluronic acid (HA), procollagen II C-propeptide (CP II), and cartilage oligomeric matrix protein (COMP). These were quantitated in ng/mL as previously described using various enzyme-linked immunosorbent assays (ELISA) as either competitive inhibition or sandwich protein binding [26]. In general, C2C, HA, and COMP levels reflect cartilage degeneration, whereas CPII levels correlate with type II collagen synthesis. All samples were analyzed in duplicate and paired at baseline, and samples from visit 3 at 3 months were run simultaneously.

### 2.4. Clinical Efficacy Variables

Four clinical variables were measured at baseline, 3, 6, and 12 months: Western Ontario and McMaster Universities Index (WOMAC) total scores, Visual Analog Pain Score (VAS 0–10), physical component score (PCS) scores on the SF-36 health survey questionnaires (physical function/bodily pain and general health), 6-min walking distance in meters (6 MWD), and US-measured SF depth (mm). The US measured depth was obtained before and after an external pneumatic compression device was inflated to 100 mmHg to facilitate aspiration by increasing available SF volumes under positive pressure [24]. 

The WOMAC score is a validated patient-self-administered index of knee osteoarthritis pain and functional capacity [27]. It consists of 24 separate items divided into three subcategories (pain, stiffness, and physical function) rated on a difficulty scale as either none, slight, moderate, very, or extreme. The PCS score is a composite score of 21 questions, which are related to four domains: physical functioning, role limitations due to physical health, pain, and general health. The maximum value of 100 would indicate no functional limitations, no pain, and excellent health scored using the following instrument: https://chiro.org/LINKS/How_to_score_the_SF-36.shtml, accessed on 5 July 2023 [28,29].

Lower values on the self-reported WOMAC scores and VAS indicate improved function or less pain, whereas higher PCS scores and greater distance on the 6 MWD indicate an improvement in function with less pain and an ability to ambulate further. We defined the subset of responders based upon OMERACT-OARSI definitions as those subjects who had either a 50% improvement in function on WOMAC scores or a 50% reduction in pain on VAS scores, or those with a 20% improvement in function on WOMAC scores and a 20% reduction in pain on VAS scores [30].

The SF depth was measured on the recorded US image (GE logiq e) as the largest anechoic region in mm of depth on either the lateral (*n* = 30) or medial (*n* = 4) infrapatellar compartment. All study data (demographics, medical history, prior treatments, screening criteria) and results were placed into the NJH REDCap^TM^ version 13.1.37 (Vanderbilt University, Nashville, TN, USA) web-based secure research database system for storage and subsequent statistical analysis.

### 2.5. Statistical Analysis

Statistical differences between baseline values and results at 3, 6, and 12 months were determined using a paired ANOVA test with *p* < 0.05 significance. SF and peripheral blood protein levels of each analyte between baseline and those at 3 months were also measured using paired ANOVA, with the cytokine concentration outcomes transformed into log10 with *p*-values < 0.05 considered significant and adjusted for the number of analytes using the Bonferroni method. If values were missing for an individual subject for a specific analyte, then that subject was excluded from statistical analysis for that specific analyte. For the purpose of calculations, samples that exceeded the upper limit of the analytical measurement range or those that were below the detection limit were assigned the upper limit value or lower limit value, respectively, for the respective cytokine, chemokine, MMP, or protein, as previously described [15].

A linear regression analysis of improvement on WOMAC scores was regressed against log10-transformed analyte concentrations to determine associations between clinical improvement and baseline cytokine concentrations. All modeling was performed in the R language [31]. Correlations were then performed to determine which baseline clinical features (age, gender, BMI, prior surgery, radiographic severity KL II vs. III, and serum or SF biomarker proteins) correlated best with IA HA responsiveness at 3, 6, and 12 months. Differences between paired serum OA biomarkers were analyzed using paired *t* tests. A non-paired *t*-test was used to compare differences in age and BMI between responders and non-responders, and Fisher’s exact test was used for the other clinical variables.

## 3. Results

**Figure 2:**
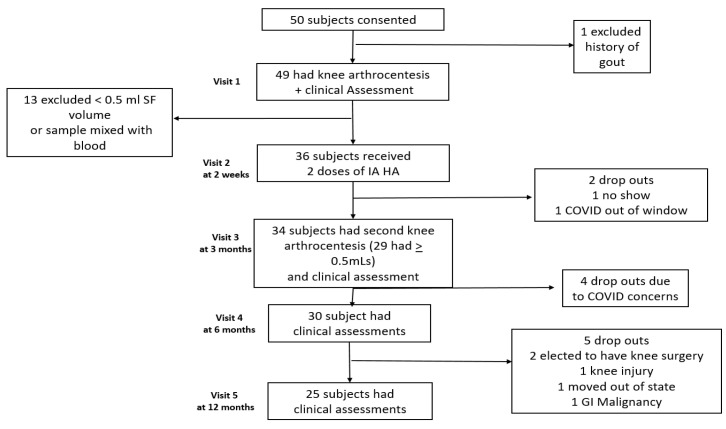
Subject participation, timeline of study visits, and reasons for withdrawals.

Thirty-six of the subjects had adequate aspirated SF volumes on their first visit and therefore received two IA HA injections and continued study enrollment. Figure 2 displays the study subject participation numbers and reasons for any withdrawals. No subjects were excluded from enrolling in this study based on the presence or absence of knee effusions on physical examination, including those with very small effusions observed on US imaging during their initial visit. Thirteen of the 49 enrolled subjects were ineligible to continue to participate since their aspirated SF volume was <0.5 mL. The average measured SF depth on US was only 3.2 ± 2.2 mm before the pneumatic device was inflated, even among those with a successful SF aspiration of ≥0.5 mL. On physical examination, only 1 of our 49 enrolled subjects had a clinically apparent effusion, whereas three others only had an effusion with a fluid bulge in the medial compartment during manual compression of the lateral compartment. Therefore, 92% of our patients had non-effusive knee OA.

The results in Table 1 display the demographic and clinical features of 34 subjects who completed baseline and 3-month evaluations. An equal number of female and male patients, who also met the BMI inclusion criteria of <40, had their 3-month follow-up visits. No study subjects were excluded based on the size of the knee effusion or prior surgical interventions, except for total knee arthroplasty. There were slightly more subjects with a KL III severity score (56%) than those with a KL II rating (44%), whereas KL I or IV scores were exclusion criteria. We enrolled twelve subjects with the following prior surgical procedures on the aspirated knees: five with meniscectomies, three with ACL reconstruction, two with surgery for recurrent patella dislocations, and two with arthroscopic resurfacing/debridement procedures. Ten of the 12 subjects who received prior surgery returned for their 3-month follow-up visit and therefore were listed in Table 1. The mean age of those with prior surgery (58 years, range 35–71) was similar to that of those without surgery (60 years, range 39–78). The higher prevalence of the lateral (88%) vs. medial (12%) site of joint aspiration reflected the preferred site by the performing physician to reduce subject discomfort unless the medial joint site had a substantially larger joint effusion on US.

The clinical efficacy and measured SF depth results for the subjects who completed baseline, 3-, 6-, and 12-month visits are displayed in Table 2. Sustained clinical and statistically significant improvements compared to baseline values were observed on WOMAC scores and self-reported VAS at 12 months, with a 50% and 54% decrease, respectively (*p* < 0.0001). PCS scores also significantly increased over baseline scores by 24% at 12 months (*p* < 0.0001). The 6 MWD improved at 3 months by 7% (*p* < 0.01); however, the improved distance walked at 6 and 12 months was not statistically significant. In a subset of 18 of the 34 patients at 3 months with an adequate SF volume remaining for analysis and without blood contamination, the SF total WBC count fell 54% at 3 months from 199 ± 200 to 92 ± 70 cells/mm^3^; however, this difference was not statistically significant (*p* = 0.05). While improvements in WOMAC, VAS, and PAS scores were observed at 3, 6, and 12 months, the measured SF volumes before or after inflation of the pneumatic compression device were slightly lower at 3 months. However, these differences did not achieve statistical significance. Only one study-related adverse event was observed (1 out of 72 injections), with one subject experiencing increased knee pain 4 h after her second IA HA injection with some knee swelling 24 h later, which resolved completely within one week, and then she was able to resume running.

Table 3 displays the demographic and clinical features of the 30 subjects who met the criteria as responders compared to the six non-responder subjects. Twenty-four respondent subjects had both a 50% improvement in WOMAC scores and a 50% reduction in pain on the VAS. Four respondent subjects met criteria by having a 50% improvement in function on WOMAC scores or a 50% reduction in pain on the VAS scale. Only two subjects met responder criteria by having a 20% improvement in function on the WOMAC and a 20% reduction in pain on the VAS scale. There were no statistical differences between responders and non-responders in baseline demographic features of age and BMI using the unpaired *t*-test or based upon gender, prior surgical intervention, or KL ratings using the Fisher’s exact test. Furthermore, no statistical differences were observed among responders vs. non-responders based upon baseline values on WOMAC, VAS, PCS score, 6 MWD, or the amount of SF measured by US.

The results of the OA serum biomarkers are presented in Table 4. The results of only those subjects with paired serum collected at baseline and 3 months later (34 of 36 subjects who received HA injections) were analyzed for statistical differences. While serum levels of C2C, CP II, and COMP were lower at 3 months compared to baseline values, these differences were not statistically significant. In contrast, HA levels were actually higher at 3 months, but those levels were also not statistically different from baseline values.

The SF levels of various protein biomarkers 3 months after IA HA injections and percentage increases or decreases from baseline values are reported in Table 5. These SF protein levels are from paired samples analyzed on the Luminex platform using identical 19-plex, 3-plex, or 5-plex kits on the same day for each subject’s paired samples. Due to the number of separate proteins analyzed and subsequent sample depletion as the assay needed to be validated during multiple prior runs, the number of paired SF samples available for final analysis was either 10 or 16 paired subjects, as reported in Table 5. While some analytes had large changes from baseline levels, including a 61% increase for IL-8 and an 87% decrease in IL-4 levels at 3 months, only the observed 16% reduction in MMP-10 levels at 3 months was statistically different from baseline values. *p*-values were calculated with a paired two-sided *t*-test on the log-concentrated levels in pg/mL.

To determine if baseline SF protein biomarker values predicted an improved response to IA HA injections at 3 months, linear regression statistics on each of these 15 biomarker protein levels in Table 5 were utilized to identify any significant correlations between baseline values from 26 separate subjects and subsequent changes in their total WOMAC scores at 3 months. No significant correlations were observed between baseline levels of any of these 15 SF proteins and changes in WOMAC scores at 3 months, with *p*-values ranging from 0.10 to 0.97.

## 4. Discussion

### 4.1. Injection and Aspiration Technique

We used US-visualized needle insertion and a pneumatic external compression device to ensure that the HA product was delivered with greater accuracy into the intra-synovial space. We also used an 18-gauge needle for aspiration since our prior study indicated that the very high SF viscosity on normal knees required a larger-bore needle for successful knee aspirations, even when performed under positive pressure using the pneumatic compression device [15]. We therefore confirmed with direct needle visualization on the US monitor that the aspirating and injecting needle was in the anechoic region. Prior studies using non-imaged guided knee IA injections indicate the intrasynovial space may be missed in 20–30% of attempts, depending upon the volume of SF and experience of the performing physician [22,32]. This error rate could be even higher among our OA patients since they had very small SF volumes measured by depth on US, with a mean of only 3.2 ± 2.2 mms. Our aspiration technique may also have facilitated a more successful aspiration of SF for biomarker analysis using a pneumatic compression device. This increases the amount of SF available after inflation, and SF is under positive pressure. In a report by Iqbal et al. using non-image-guided aspiration in a flexed knee aspiration technique in patients without large effusions, they were able to increase the successful knee aspiration rate from 41% to 75% using a pneumatic thigh cuff inflated to 100 mmHg [33]. Therefore, our ability to aspirate ≥ 0.5 mL in 74% of our patients on their initial visit probably reflects the benefit of utilizing the US-guided needle visualization technique with external compression [34].

The difference in our injection technique compared to landmarked guided injections might also explain the very low incidence of injection site product reactions (only 1 of 72 HA injections) and provide an explanation for the longer clinical benefit durability of 12 months. These durable clinical improvements occurred after a single series of two HA injections 7 days apart. Bisicchia et al. also reported clinical benefit in a prospective randomized study of IA HYADD4 at 26 weeks on WOMAC and VAS, which was superior to IA methylprednisolone; however, benefit above baseline scores was not observed at 52 weeks for either product using a non-image-guided injection technique [35]. In another knee OA study using the same IA HA product, Benazzo et al. reported improvements in WOMAC scores at 6 months in a prospective open-label study from a single series of two IA injections, and clinical benefit was maintained at 52 weeks following a repeat series of IA injections at 6 months without image guidance [36]. In a retrospective report using the ANTIAGE registry of clinicians who performed IA injections of HYADD4 using ultrasound on KL II-IV knee OA patients, Priano reported significant reductions in WOMAC and VAS scores among 74.5% of patients (698 of 937) at 6 months [21]. At 12 months, improvement in pain at rest and with movement was also reported as the only clinical outcome data available from 11% of patients (106 of 937) available for analysis.

Our observed clinical efficacy results and durability of response may also have been related to this specific HA product. In a meta-analysis of low vs. high molecular weight IA HA injection products, Hummer et al. reported clinically important improvements in pain reduction when high but not low molecular weight HA injection products were injected compared with placebo [37]. A review by Ferkel et al. also supports molecular weight and other differences in product manufacturing and composition as important factors in efficacy outcomes in clinical trials using different HA products [38].

### 4.2. SF Volume Measurements

Even though we observed significant improvements in WOMAC, VAS, and PCS scores at 3, 6, and 12 months, this was not associated with statistically significant changes in the amount of SF as determined by the measured depth of the anechoic region on US either before or after inflation of the external pneumatic compression device. Results of the Multicenter Osteoarthritis Knee Hyaluronic Acid Study (MOKHA) included 46 knee OA patients with very similar demographics as our study subjects regarding age and KL rating who also received IA HYADD4 injections [39]. They recorded an 18% reduction in knee effusions on MRI imaging at 6 months but not at 12 months. They also reported clinical benefit at 6 and 12 months on Knee Injury and Osteoarthritis Outcome Scores (KOOS); however, their subjects received a second series of IA HA injections at 6 months.

In another very ambitious prospective placebo-controlled study, McAlindon et al. performed a double-blinded knee OA efficacy study of normal saline vs. triamcinolone injections every 3 months for 2 years. They also reported no change in SF volumes quantitated on MRI at 2 years between these two groups or from baseline values [40]. They also reported no differences in functional outcomes between the two groups, despite the greater cartilage volume loss reported in the triamcinolone group. However, the suggestion that corticosteroids cause additional cartilage loss and thus progression of osteoarthritis has not been substantiated in other careful radiographic progression trials. Several studies have demonstrated no difference in the gold standard of radiographic progression of OA or cartilage turnover biomarkers between intra-articular corticosteroid treatment and placebo or intra-articular HA administration [41,42,43,44].

### 4.3. Serum and SF Biomarkers

Posey et al. have reviewed the role of serum COMP levels in various forms of arthritis [45]. Since levels are elevated in early but not advanced OA, we anticipated a fall in levels at 3 months following IA HYADD4 injections since all of our patients were KL II or III. They also reviewed the effect of weight-bearing exercise, which increases COMP levels; therefore, it is possible our observed 27% increase in levels at 3 months may have been related to an increase in ambulation related to less pain as documented on WOMAC, VAS, and PCS scores. We also documented an increase in 6 MWD at 3 months, as noted in Table 2. Our baseline and 3-month levels of COMP and C2C might have been higher than reported in other studies since our samples were obtained within 45 min of subjects completing their 6 MWD. While elevated serum HA levels correlate with the progression and severity of OA, we are not aware of serum HA measurements before and after IA HA injection [46].

Synovial fluid is potentially an ideal source of biomarkers to investigate the pathogenic mechanisms of cartilage damage in OA, characterize different disease endotypes, and identify potential therapeutic targets. SF biomarkers may also identify those at highest risk for disease progression as well as those most likely to respond to a specific therapeutic agent. SF is an ultrafiltrate of plasma due to the lack of a typical basement membrane within synovial tissue, and in OA, various inflammatory and catabolic proteins are released by synoviocytes. Since cartilage does not have a blood supply, the SF also provides nutritional support, and synoviocytes produce key SF proteins that help maintain cartilage health and preserve function, such as HA and lubricin, as well as proteinases, collagenases, and prostaglandins [47].

SF was available for SF biomarker analysis in a much smaller subset of our study subjects than planned, as only 18 of our 34 subjects had paired samples at 3 months since some subjects had smaller volumes at 3 months compared to their baseline visit, which made aspiration more difficult. Some of the patients with aspirated volumes of 0.5 to 1.0 mL were also contaminated with blood as determined by visual inspection and the SF WBC count and differential, and some were consumed during feasibility studies. We have observed from multiple prior SF quantitative protein assays on patients with various forms of knee arthritis that the accuracy of SF cytokine levels was not robust on SF volumes < 1.0 mL or when contaminated with peripheral blood arising from intra-synovial bleeding during needle insertion [15]. Therefore, we were only able to report results from a subset of 16 and 10 patients who had paired baseline and 3-month samples in Table 5. Furthermore, the high viscosity of our SF sample matrix made sample processing and analysis, particularly with the Luminex multiplex bead arrays, challenging. Sample viscosity may have contributed to clogging of the Luminex fluidics system and poor bead recovery, therefore leading to uninterpretable results for some samples.

We observed that only 1 of 15 synovial fluid proteins (MMP 10 levels) had significant changes at 3 months following IA HA, even though the clinical improvement on WOMAC, VAS, PCS scores, and 6 MWD at this time was significant. Barksby et al. report that MMP 10 (also called stromelysin 2) is produced by synovial fibroblasts and articular chondrocytes and is expressed in diseased joint synovium, suggesting it is also an activator of procollagenases [48]. They also reported similar SF MMP-10 levels in patients with OA compared to those with rheumatoid arthritis and juvenile idiopathic arthritis. Therefore, a reduction in MMP-10 levels 3 months after IA HA suggests one potential mechanism of clinical efficacy if this protease is a major contributor to cartilage collagenolysis in OA. However, the roles of various MMPs in OA pathogenesis are very complex, and levels are also modulated by various cytokines and proteins within the SF, bone, and cartilage, as reviewed by Mehana and colleagues [49].

Our study with a small number of SF samples may have been underpowered to identify statistically different changes in some of these SF proteins and other biomarkers of inflammation. It is also possible that clinical improvement mediated by IA HA injections may result in changes in the inflammatory or catabolic proteins in cartilage that occur earlier than 3 months. Falcinelli et al. documented lower SF levels of IL-6, MMP-2, and MMP-13 when measured 7 days after IA administration of the same HA product (HYADD 4-G) [50]. It is also likely that the 54% lower SF WBC counts observed at 3 months might have achieved statistically significant differences if our sample size were larger.

In another study reporting SF protein biomarkers after a different IA HA product (1% sodium hyaluronate), after three weekly injections on 28 subjects, there was a greater reduction in SF TNF alpha levels at 6 months in adults < 65 years of age compared to adults > 65 years of age. However, the other inflammatory cytokines (IL-1 beta, IL-6, IL-8, and IL-12) and levels of IL-4, IL-10, IL-13, and monocyte chemotactic did not differ from baseline values [51]. In another interesting study of three weekly IA Hyaluronan injections in OA patients with clinical knee effusions, one week post-treatment, IL-6 levels were statistically lower in both normal saline-injected (*n* = 19) and HA-treated patients (*n* = 22), whereas TNF alpha and IL-8 levels did not change in either group [52].

Our open-label, non-blinded study did not include a placebo injection because the goal was not to determine the efficacy of IA HA compared to saline injection. Our objective was to determine if observed clinical benefit at 3, 6, or 12 months was associated with reductions in the amount of measured knee SF volumes or changes in SF WB counts, serum cartilage, and SF biomarkers at 3 months. We also wanted to identify if any baseline clinical and demographic features or SF biomarker profiles predicted clinical responsiveness to IA HA injections at 3 months. In a meta-analysis of 149 efficacy trials of various modes of delivery of placebo, Bannuru et al. described that the greatest placebo effect occurred when the placebo was delivered via an intra-articular route, but they also acknowledged the potential for therapeutic benefit from saline injections by diluting inflammatory mediators [53]. A reduction in SF IL-6 levels one week post-saline injection suggests a potential therapeutic benefit from knee aspiration or normal saline instillation into the intra-synovial space rather than exclusively a “placebo effect”, since saline and HA-injected subjects had improved WOMAC scores, but higher values were observed in the IA HA-treated group [54]. Altman et al. reviewed the evidence in a meta-analysis of 38 randomized controlled trials and concluded that, given the substantial and uniform reduction of OA knee pain using IA saline, a potential therapeutic effect rather than solely due to a placebo effect may well account for some of the observed clinical improvement [55].

Our OA study population would have been more homogeneous if we had excluded those patients with prior knee surgery. However, since these are common orthopedic procedures among knee OA patients who receive therapeutic IA HA injections, they were not excluded. The overall mean age of the previously operated subjects was similar to that of the non-operated group, as noted in Table 1. Our inclusion of these subjects suggests that symptomatic knee OA, even among those with prior non-arthroplasty surgical procedures, may also display a durable response to this IA HA product when delivered via US guidance.

### 4.4. Strengths

The single-center study design ensured uniform adherence to study protocol and aspiration/injection technique, as well as SF sample handling, compared to a multicenter study. This is important since some of these cytokines are labile at room temperature and sensitive to degradation, whereas all of our samples were centrifuged and cryopreserved within 60 min of collection [56]. US guidance also allowed us to perform IA HA injections more accurately among OA patients with small effusions, unlike those reported by Sezgen with palpable effusions, which may represent a different OA endotype than our study patients [52]. It is also possible that our improved durability and clinical efficacy were also due to the removal of catabolic SF proteins prior to the first of two IA HA injections, as aspiration prior to glucocorticoid injections has a therapeutic benefit [54,55].

### 4.5. Study Limitations

We acknowledge that our small sample size and the small subset of SF samples available for final analysis at 3 months may have resulted in the lack of statistically significant changes in some protein biomarkers. While all 36 subjects had ≥0.5 mL of SF aspirated on their first study visit, 3 months later, many subjects had even smaller SF volumes, and therefore some of these subjects did not have adequate SF volumes for paired SF analysis.

It is also possible that those study subjects who dropped out between their 3-, 6-, or 12-month visits may have skewed the results in favor of a higher level of responders who remained in the study at 12 months than if there were fewer voluntary withdrawals. However, despite institutional COVID restrictions and subject hesitancy due to COVID, only 4 of 34 (12%) enrolled subjects at 3 months did not return at 6 months. The two athletic subjects who dropped out between 6 and 12 months decided to pursue elective orthopedic surgical interventions, whereas three others were unable to participate due to an unrelated sports injury, malignancy, or relocation out of state. Since there were only two withdrawals due to potential lack of continued efficacy, we suspect their inclusion in our final analysis would likely not have skewed the results since the differences were *p* < 0.0001 at 12 months on the WOMAC, PCS, and VAS efficacy scales.

## 5. Conclusions

While improvements in WOMAC, VAS scores, and PCS scores on the SF 36 were observed at 3, 6, and 12 months after US-guided knee injections with this HA product, a statistically significant reduction in US-measured SF volumes was not observed at these same time points. The 6 MWD improved at 3 months but not at 6 or 12 months. IA injections using US needle visualization confirmed that the product was delivered into the intra-synovial fluid space with improved accuracy, which may also have resulted in our very low incidence of observed post-IA injection reactions (1 out of 72 injections) as well as a greater durable clinical response lasting 12 months. Our baseline clinical features and SF biomarker panel did not predict responders vs. non-responders. A fall in MMP-10 levels was observed at 3 months, whereas the other fourteen SF proteins and four serum biomarkers were unchanged.

The sustained efficacy results in this study may not be comparable to IA injections using a different HA product if injected without US guidance or without an aspiration prior to the first HA injection. The current recommendation against the use of IA HA for knee osteoarthritis by the American College of Rheumatology and the American Academy of Orthopedic Surgeons reflects the small effect size of prior studies that delivered HA without image guidance [57,58,59]. In Europe, however, the 2020 EULAR recommendations support the use of IA HA injections for knee OA [60]. In addition, our results may not be generalizable to different OA phenotypes, including those with morbid obesity, more advanced KL grades, or large knee effusions.

We anticipate a larger study will be necessary to validate a SF-based biomarker panel to identify which individual OA patients will most likely receive the greatest benefit from HA or therapeutic IA injections. This information may also help identify if these agents reduce catabolic pro-inflammatory proteins, which cause irreversible cartilage loss, and discover more effective therapeutic targets to improve the quality of life for patients with knee OA.

## Figures and Tables

**Table 1:** Clinical features and demographic information on 34 OA subjects who completed baseline and 3-month visits following intra-articular HA knee injections.

Age	60.8 years (35–78)
Gender	17 Male (50%)/17 Female (50%)
BMI kg/m^2^	28 (20–39)
Prior knee surgery	10 (29%)
K-L grade	
II	15 (44%)
III	19 (56%)
Knee injected	
Right	18 (53%)
Left	16 (47%)
Lateral	30 (88%)
Medial	4 (12%)

**Table 2:** Subject’s mean and standard deviation values with percentage change in four functional and pain instruments and synovial fluid depth measurements at baseline compared to values 3-, 6-, and 12-months post-IA HYADD4 injections with statistical significance in *p*-values.

	Baseline Mean ± SD *n* = 36	3-Month Mean ± SD *n* = 34	6-Month Mean ± SD *n* = 30	12-Month Mean + SD *n* = 25
**WOMAC score**	771 ± 394	463 ± 358	464 ± 352	402 + 333
		40% decrease *p* < 0.0001	40% decrease *p* < 0.0001	50% decrease *p* < 0.0001
**VAS score**	4.9 ± 2.0	2.7 ± 1.7	2.4 ± 1.9	2.2 + 1.73
		45% decrease *p* < 0.0001	51% decrease *p* < 0.0001	54% decrease *p* < 0.0001
**PCS score**	64.7 ± 18.1	74.6 ± 18.7	76.5 ± 18.1	81.2 + 11.9
		15% increase *p* < 0.0001	18% increase *p* < 0.0001	24% increase *p* < 0.0001
**6 MWD-**	404 ± 67	432 ± 83	422 ± 75	424 + 69
**Meters**		7% increase *p* < 0.007	5% increase NS	5% increase NS
**SF before**	3.2 ± 2.2	3.1 ± 2.2	4.0 ± 2.9	4.2 +2.8
**inflation (mm)**		3% decrease NS	25% increase NS	31% increase NS
**SF after**	6.4 ± 3.7	5.2 ± 2.8	7.5 ± 4.0	7.5 + 3.4
**inflation (mm)**		18% decrease NS	17% increase NS	17% increase NS

Legend: WOMAC score = Western Ontario and McMaster Universities Osteoarthritis Index; VAS = Visual Analog Pain score; PCS score = subset on SF 36 quality of life questionnaire; 6 MWD = 6-min walking distance; SF = depth of synovial fluid measured on ultrasound; NS = not significant values compared to baseline.

**Table 3:** Clinical features, functional status, pain, and synovial fluid measurements at baseline between responders and non-responders with *p*-values.

Demographics	Responders *n* = 30	Non Responders *n* = 6	*p*-Values
Age mean and range	59 (19–78) years	62 (54–78) years	0.48
Gender and %	13 F (43%)/17 M (57%)	4 F (66%)/2 M (33%)	0.39
BMI mean and range	28.1 (19–39)	26.6 (21–32)	0.61
Prior Surgery and %	9 (30%)	3 (50%)	0.38
KL II or III and %	18 KL II (60%)/12 KL III (40%)	5 KL II (83%)/1 KL III (17%)	0.39
**Functional Status, pain and US depth of SF**			
WOMAC score	814	675	0.44
VAS (1–10 scale)	5.2	3.5	0.051
PCS score	64.1	64.6	0.96
6 MWD (meters)	406	428	0.49
US depth			
Before inflation	3.7 mm	4.3 mm	0.75
After inflation	7.7mm	5.9 mm	0.37

**Table 4:** Serum cartilage biomarker levels at baseline and 3 months after IA HA injection with *p*-values.

	Baseline Mean ± SD	3-Month Mean ± SD	% Increase or Decrease from Baseline	*p*-Values
	*n* = 34	*n* = 34		
C2C ng/mL	278 ± 48	263 ± 52	5% decrease	0.08
COMP ng/mL	828 ± 400	798 ± 435	4% decrease	0.36
HA ng/mL	41 ± 29	52 ± 58	27% increase	0.27
CPII ng/mL	1269 ± 508	1204 ± 549	5% decrease	0.32

Legend: C2C = collagen Type II cleavage product; COMP = cartilage oligomeric matrix protein; HA = hyaluronic acid; CPII = procollagen II C-Propeptide. *p*-values were calculated using paired *t*-tests.

**Table 5:** The mean values, standard deviation, percentage change, and *p*-values for each analyte at baseline and 3 months after IA HA injections.

Protein	Baseline Mean ± SD pg/mL	3-Month Mean ± SD pg/mL	% Increase or Decrease from Baseline	*p*-Values
IL-1ra *n* = 16	345 ± 332	518 ± 564	50% increase	0.127
IL-4 *n* = 10	1971 ± 5243	251 ± 107	87% decrease	0.395
IL-6 *n* = 16	60 ± 98	40 ± 44	33% decrease	0.905
IL-7 *n* = 16	7 ± 1	8 ± 2	14% increase	0.167
IL-8 *n* = 16	36 ± 41	58 ± 77	61% increase	0.273
IL-15 *n* = 16	6 ± 3	7 ± 4	17% increase	0.825
IL-18 *n* = 16	109 ± 63	103 ± 63	6% decrease	0.402
IGFBP-1 *n* = 16	6376 ± 9346	6707 ± 12,560	5% increase	0.406
IGFBP-3 *n* = 16	36,517 ± 49,159	40,790 ± 58,870	12% increase	0.808
MMP-1 *n* = 10	7971 ± 6827	8323 ± 9046	4% increase	0.541
MMP-2 *n* = 10	283,599 ± 218,875	249,307 ± 194,342	12% decrease	0.325
MMP-3 *n* = 10	245,119 ± 153,269	235,275 ± 175,421	4% decrease	0.293
MMP-8 *n* = 10	1354 ± 503	1729 ± 1899	28% increase	0.686
MMP-9 *n* = 10	5014 ± 4464	6375 ± 9225	27% increase	0.956
MMP-10 *n* = 16	238 ± 206	200 ± 208	16% decrease	0.0427

Legend: IL-1 ra = Interleukin 1 receptor antagonist, IL-4 = Interleukin 4, IL-6 = Interleukin 6, IL-7 = Interleukin 7, IL-8 = Interleukin 8, IL-18 = Interleukin 18, IL-15 = interleukin 15, IGFBP-1 Insulin-Like Growth Factor Binding Protein 1, IGFBP-3 = Insulin-Like Growth Factor Binding Protein 3, MMP-1 Matrix Metalloproteinase 1, MMP-2 = Matrix Metalloproteinase 2, MMP-3 = Matrix Metalloproteinase 3, MMP-8 = Matrix Metalloproteinase 8, MMP-9 = Matrix Metalloproteinase 9, MMP-10 = Matrix Metalloproteinase 10. *p*-values < 0.5 was only observed for MMP 10 levels.

## Data Availability

The data used to generate Figure 1, Table 1, Table 2 and Table 3 were entered into REDCap database at National Jewish Health and contains personal health information (PHI) so is considered confidential except for access by the investigators and biostatistician James Crooks who performed the statistical analysis.

## References

[1] Katz J.N., Arant K.R., Loeser R.F. (2021). Diagnosis and Treatment of hip and knee osteoarthritis A Review. JAMA.

[2] US Bureau of Labor Statistics Consumer Price Index for All Urban Consumers (CPI-U): U.S. City Average by Expenditure Cate-gory. https://www.bls.gov/news.release/cpi.t01.htm.

[3] Schieker M., Conaghan P.G., Mindeholm L., Praestgaard J., Solomon D.H., Scotti C., Gram H., Thuren T., Roubenoff R., Ridker P.M. (2020). Effects of interleukin-1β inhibition on incident hip and knee replacement: Exploratory analyses from a randomized, double-blind, placebo-controlled trial. Ann. Intern. Med..

[4] Heijman M.W.J., Fiolet A.T.J., Mosterd A., Tijssen J.G.P., van den Bemt B.J.F., Schut A., Eikelboom J.W., Thompson P.L., van den Ende C.H.M., Nidorf S.M. (2023). Association of low-dose colchicine with incidence of knee and hip replacements. Ann. Intern. Med..

[5] Zhu K.Y., Acuna A.J., Samuel L.T., Grits D., Kamath A.F. (2022). Hyaluronic acid injections for knee osteoarthritis, has utilization among Medicare beneficiaries changed between 2021 and 2018?. J. Bone Jt. Surg. Am..

[6] Loeser R.F., Goldring S.R., Scanzello C.R., Goldring M.B. (2012). Osteoarthritis: A disease of the joint as an organ. Arthritis Rheum..

[7] Ene R., Sinescu R.D., Ene P., Cirstoiu M.M., Cirstoiu F.C. (2015). Synovial inflammation in patients with different stages of knee osteoarthritis. Rom. J. Morphol. Embryol..

[8] Benito M.J., Veale D.J., FitzGerald O., van den Berg W.B., Bresnihan B. (2005). Synovial tissue inflammation in early and late osteoarthritis. Ann. Rheum. Dis..

[9] Roemer F.W., Guermazi A., Felson D.T., Niu J., Nevitt M.C., Crema M.D., Lynch J.A., Lewis C.E., Torner J., Zhang Y. (2011). Presence of MRI-detected joint effusion and synovitis increases the risk of cartilage loss in knees without osteoarthritis at 30 months follow up: The MOST study. Ann. Rheum. Dis..

[10] Wojdasiewicz P., Poniatowski L.A., Szukiewicz D. (2014). The Role of inflammatory and anti-inflammatory cytokines in the pathogenesis of osteoarthritis. Mediat. Inflamm..

[11] Goldring M.B., Otero M., Plumb D.A., Dragomir C., Favero M., El Hachem K., Hashimoto K., Roach H.I., Olivotto E., Borzi R.M. (2011). Roles of inflammatory and anabolic cytokines in cartilage metabolism: Signals and multiple effectors converge upon MMP-13 regulation in osteoarthritis. Eur. Cell Mater..

[12] Mobasheri A., Bay-Jensen A.C., van Spil W.E., Larkin J., Levesque M.C. (2017). Osteoarthritis year in review 2016: Biomarkers (biochemical markers). Osteoarthr. Cartil..

[13] Bay-Jenson A.C., Thudium C.S., Mobasheri A. (2018). Development and use of biochemical markers in osteoarthritis: Current update. Cur. Opin. Rheumatol..

[14] Lotz M., Martel-Pelletier J., Christiansen C., Brandi M.L., Bruyere O., Chapurlat R., Collette J., Cooper C., Giacovelli G., Kanis J.A. (2014). Republished: Value of biomarkers in osteoarthritis: Current status and perspectives. Postgrad. Med. J..

[15] Meehan R.T., Regan E.A., Hoffman E.D., Wolf M.L., Gill M.T., Crooks J.L., Parmar P.J., Scheuring R.A., Hill J.C., Pacheco K.A. (2021). Synovial fluid cytokines, chemokines and MMP levels in osteoarthritis patients with knee pain display a profile similar to many Rheumatoid arthritis patients. J. Clin. Med..

[16] Nelson A.E. (2018). Osteoarthritis year in review 2017: Clinical. Osteoarthr. Cartil..

[17] Altman R.D., Mango A., Forefinger A., Nazi F., Nicholls M. (2015). The mechanism of action for hyaluronic acid treatment in the osteoarthritic knee: A systemic review. BMC Musculoskelet. Discord..

[18] Altman R., Beda A., Mango A., Nazi F., Shaw P., Meese P. (2019). Anti-Inflammatory effects of intra-articular hyaluronic acid: A systemic review. Cartilage.

[19] Moreland L.W. (2003). Intra-articular hyaluronic (hyaluronic acid) in Humans? For treatment of osteoarthritis: Mechanisms of action. Arthritis Res. Ther..

[20] Bonnevie E.D., Galesso D., Secchieri C., Bonassar L.J. (2019). Frictional characterization of injectable hyaluronic acid is more predictive of clinical outcomes than traditional rheological or viscoelastic characterization. PLoS ONE.

[21] Priano F. (2017). Early efficacy of intra-articular HYADDR4 (HymovisR) injections for symptomatic knee osteoarthritis. Joints.

[22] Berkoff D.J., Miller L.E., Block J.E. (2012). Clinical utility of ultrasound guidance for intra-articular knee injections: A review. Clin. Interv. Aging.

[23] Wu T., Dong Y., Song H.x., Fu Y., Li J.H. (2016). Ultrasound-guided versus landmark in knee arthrocentesis: A systemic review. Semin. Arthritis Rheum..

[24] Meehan R., Wilson C., Hoffman E., Altimier L., Kaessner M., Regan E.A. (2019). Ultrasound measurement of knee synovial fluid during external pneumatic compression. J. Orthop. Res..

[25] Kellgren J.H., Lawrence J.S. (1957). Radiological assessment of osteo-arthrosis. Ann. Rheum. Dis..

[26] Kraus V.B., Collins J.E., Hargrove D., Losina E., Nevitt M., Katz J.N., Wang S.X., Sandell L.J., Hoffman S.C., Hunter D.J. (2017). Predictive validity of biochemical biomarkers in knee osteoarthritis: Data from the FNIH OA biomarkers consortium. Ann. Rheum. Dis..

[27] Bellamy N., Buchanan W.W., Goldsmith C.H., Campbell J., Stitt L.W. (1988). Validation study of WOMAC: A health status instrument for measuring clinically important patient relevant outcomes to antirheumatic drug therapy in patients with osteoarthritis of the hip or knee. J. Rheumatol..

[28] Ware J.E., Snow K., Klosinski M., Gandek B. (1993). SF 36 Health Survey.

[29] Patel A.A., Donegan D., Albert T. (2007). The 36-Item Short. J. Am. Acad. Orthop. Surg..

[30] Pham T., van der Heijde D., Altman R.D., Anderson J.J., Bellamy N., Hochberg M., Simon L., Strand V., Woodworth T., Dougados M. (2004). OMERACT-OARSI Initiative: Osteoarthritis Research Society International set of responder criteria for osteoarthritis clinical trials revisited. OsteoArthritis Cartil..

[31] R Core Team (2020). R: A Language and Environment for Statistical Computing.

[32] Maricar N., Parkes M.J., Callaghan M.J., Felson D.T., O’Neill T.W. (2013). Where and how to inject the knee—A systemic review. Semin. Arthritis Rheum..

[33] Iqbal A., Brahmabhatt S., Muruganandam M., Trost J.R., Farshami F.J., Cisneros D.R., Kiani A.N., McElwee M.K., Hayward W.A., Haseler L.J. (2022). Extraction of Synovial fluid from the non-effusive pathologic knee with pneumatic compression. Authorea.

[34] Bhavsar T.B., Sibbitt W.L., Band P.A., Cabacungan R.J., Moore T.S., Salayandia L.C., Fields R.A., Kettwich S.K., Roldan L.P., Emil N.S. (2018). Improvement in diagnostic and therapeutic arthrocentesis via constant compression. Clin. Rheumatol..

[35] Bisicchia S., Bernardi G., Tudisco C. (2016). HYADD4 versus methylprednisolone acetate in symptomatic knee osteoarthritis: A single-centre single blind prospective randomized controlled clinical study with a 1 year follow up. Clin. Exp. Rheumatol..

[36] Benazzo F., Perticarnini L., Padolino A., Castelli A., Gifuni P., Lovato M., Manzini C., Giordan N. (2016). A multi-centre, open label, long-term follow-up study to evaluate the benefits of a new viscoelastic hydrogel (HymovisR) in the treatment of knee osteoarthritis. Eur. Rev. Med. Pharmacol. Sci..

[37] Hummer C.D., Angst F., Ngai W., Whittington C., Yoon S.S., Duarte L., Manitt C., Schemitsch E. (2020). High molecular weight intraarticular hyaluronic acid for the treatment of knee osteoarthritis: A network meta-analysis. BMC Musculoskelet. Disord..

[38] Ferkel E., Manjoo A., Martins D., Bhandari M., Sethi P., Nicholls M. (2023). Intra-articular Hyaluronic acid treatments review of product properties. Cartilage.

[39] Henrotin Y., Bannuru R., Malaise M., Ea H.K., Confavreux C., Bentin J., Urbin-Choffray D., Conrozier T., Brasseur J.P., Thomas P. (2019). Hyaluronan derivative HYMOVISR increases cartilage volume and Type II collagen turnover in osteoarthritic knee: Data from MOKHA study. BMC Musculoskelet. Disord..

[40] McAlindon T.E., LaValley M.P., Harvey W.F., Price L.L., Driban J.B., Zhang M., Ward R.J. (2017). Effect of Intra-articular triamcinolone vs saline on knee cartilage volume and pain in patients with knee osteoarthritis, a randomized clinical trial. JAMA.

[41] Latourte A., Rat A.C., Omorou A., Ngueyon-Sime W., Eymard F., Sellam J., Roux C., Ea H.K., Cohen-Solal M., Bardin T. (2022). Do glucocorticoid injections increase the risk of knee osteoarthritis progression over 5 Years?. Arthritis Rheumatol..

[42] Bucci J., Chen X., LaValley M., Nevitt M., Torner J., Lewis C.E., Felson D.T. (2022). Progression of knee osteoarthritis with use of intraarticular glucocorticoids versus hyaluronic acid. Arthritis Rheumatol..

[43] Klocke R., Levasseur K., Kitas G.D., Smith J.P., Hirsch G. (2018). Cartilage turnover and intra-articular corticosteroid injections in knee osteoarthritis. Rheumatol. Int..

[44] Raynauld J.P., Buckland-Wright C., Ward R., Choquette D., Haraoui B., Martel-Pelletier J., Uthman I., Khy V., Tremblay J.L., Bertrand C. (2003). Safety and efficacy of long-term intraarticular steroid injections in osteoarthritis of the knee: A randomized, double-blind, placebo-controlled trial. Arthritis Rheum..

[45] Posey K.L., Hecht J.T. (2008). The role of cartilage oligomeric matrix protein (COMP) in skeletal disease. Curr. Drug Targets.

[46] Sasaki E., Tsuda E., Yamamoto Y., Maeda S., Inoue R., Chiba D., Fujita H., Takahashi I., Umeda T., Nakaji S. (2015). Serum hyaluronic acid concentration predicts the progression of joint space narrowing in normal knees and established knee osteoarthritis—A five year prospective cohort study. Arthritis Res. Ther..

[47] Nygaard G., Firestein G.S. (2020). Restoring synovial homeostasis in rheumatoid arthritis by targeting fibroblast-like synoviocytes. Nat. Rev. Rheumatol..

[48] Barksby H.E., Milner J.M., Patterson A.M., Peak N.J., Hui W., Robson T., Lakey R., Middleton J., Cawston T.E., Richards C.D. (2006). Matrix Metalloproteinase 10 promotion of collagenolysis via procollagenase activation. Arthritis Rhem..

[49] Mehana E.S.E., Khafaga A.F., El-Blehi S.S. (2019). The role of matrix metalloproteinases in osteoarthritis pathogenesis: An updated review. Life Sci..

[50] Falcinelli E., Giordan N., Luccioli F., Piselle E., La Paglia G.M.C., Momi S., Mirabelli G., Petito E., Alunno A., Gresele P. (2020). Randomized Trial of HymovisR versus SynviscR on Matrix Metalloproteinase in Knee osteoarthritis. Muscles Ligaments Tendons J..

[51] Vincent H.K., Percival S.S., Conrad B.P., Seay A.N., Montero C., Vincent K.K. (2013). Hyaluronic acid (HA) Viscosupplementation on synovial fluid inflammation in knee osteoarthritis: A Pilot study. Open Orthop. J..

[52] Sezgin M., Demirel A.C., Karaca C., Ortancil O., Ulkar G.B., Kanik A., Cakci A. (2005). Does Hyaluronan affect inflammatory cytokines in knee osteoarthritis?. Rheumatol. Int..

[53] Bannuru R.R., McAlindon T.E., Sullivan M.C., Wong J.B., Kent D.M., Schmid C.H. (2015). Effectiveness and implications of alternative placebo treatments, a systemic review and network meta-analysis of osteoarthritis trials. Ann. Intern. Med..

[54] Weitoft T., Uddenfeld P. (2000). Importance of synovial fluid aspiration when injecting intra-articular corticosteroids. Ann. Rheum. Dis..

[55] Altman R.D., Devji T., Bhandari M., Fierlinger A., Niazi F., Christensen R. (2016). Clinical benefit of intra-articular saline as a comparator in clinical trials of knee osteoarthritis treatments: A systemic review and meta-analysis of randomized trials. Semin. Arthritis Rheum..

[56] Knight V., Long T., Meng Q.H., Linden M.A., Roads D.D. (2020). Variability in the Laboratory Measurement of Cytokines: A Longitudinal Summary of a College of American Pathologists Proficiency Testing Survey. Arch. Pathol. Lab. Med..

[57] Kolasinski S.L., Neogi T., Hochberg M.C., Oatis C., Guyatt G., Block J., Callahan L., Copenhaver C., Dodge C., Felson D. (2020). 2019 American College of Rheumatology/Arthritis Foundation guideline for the management of osteoarthritis of the hand, hip, and knee. Arthritis Rheumatol..

[58] Jevsevar D.S., Brown G.A., Jones D.L., Matzkin E.G., Manner P.A., Mooar P., Schousboe J.T., Stovitz S., Sanders J.O., Bozic K.J. (2013). American Academy of Orthopaedic Surgeons evidence-based guideline on treatment of osteoarthritis of the knee, 2nd edition. J. Bone Jt. Surg. Am..

[59] Johansen M., Bahrt H., Altman R.D., Bartels E.M., Juhl C.B., Bliddal H., Lund H., Christensen R. (2016). Exploring reasons for the observed inconsistent trial reports on intra-articular injections with hyaluronic acid in the treatment of osteoarthritis: Meta-regression analyses of randomized trials. Semin. Arthritis Rheum..

[60] Pendleton A., Arden N., Dougados M., Doherty M., Bannwarth B., Bijlsma J., Cluzeau F., Cooper C., Dieppe P., Gunther K. (2000). EULAR recommendations for the management of knee osteoarthrtitis: A report of a task force of the standing committee for the international clinical studies including therapeutic trials (ESCISIT). Ann. Rheum. Dis..

